# MicroRNA-181a regulates Treg functions via TGF-β1/Smad axis in the spleen of mice with acute gouty arthritis induced by MSU crystals

**DOI:** 10.1590/1414-431X2022e12002

**Published:** 2022-12-02

**Authors:** Yu Wang, Shenghao Tu, Ying Huang, Kai Qin, Zhe Chen

**Affiliations:** 1 Department of Integrated Traditional Chinese and Western Medicine, Tongji Hospital, Tongji Medical College, Huazhong University of Science and Technology, Wuhan, Hubei, China

**Keywords:** Regulatory T cell, miR-181a, Transforming growth factor-β1, Sepsis

## Abstract

Regulatory T cells (Tregs) play critical roles in restricting inflammatory pathogenesis and limiting undesirable Th2 response to environmental allergens. However, the role of miR-181a in regulating acute gouty arthritis (AGA) and Treg function remains unclear. This study aimed to investigate the potential roles of miR-181a in Treg immunity and the associated signaling pathway in the AGA mouse model. A solution with monosodium urate (MSU) crystals was injected into the joint tissue of mice to induce AGA. ELISA was used to examine inflammatory factors in blood samples, and flow cytometry was used to analyze Treg profile in mice with MSU-induced AGA. Cell proliferation and viability were assessed by CCK-8 assay. TGF-β1/Smad signaling activation was detected by western blot. We found that miR-181a expression showed a positive correlation with the changes of splenic Tregs percentage in AGA mice. miR-181a regulated the TGF-β1/Smad axis, since the transfection of miR-181a mimic increased the level of TGF-β1 and the phosphorylation of Smad2/3 in Tregs in AGA mice. Additionally, miR-181a mimic also promoted responses of Tregs via TGF-β1 *in vitro* and *in vivo*. Our work uncovered a vital role of miR-181a in the immune function of Treg cells by mediating the activity of the TGF-β1/Smad pathway in the AGA mouse model induced by MSU.

## Introduction

The pathogenesis of acute gouty arthritis (AGA) is associated with the imbalance between Th1 and Th2 cell subsets, as well as the undesirable response of Th2 to environmental allergens (1). In arthritis, localized Th1 over-activation can lead to inflammatory response and tissue damage (2). Strategies targeting Th1 and Th2 imbalance have been shown to alleviate rheumatoid arthritis in mouse models and human patients. For example, targeting adrenergic receptors shifts T helper (Th) cytokines from a Th1 to Th2 profile in immune organs and attenuates arthritis in mice (3). Inhibiting histone deacetylase (HDAC) activity by trichostatin suppresses Th1 response and protects against collagen-induced rheumatoid arthritis in mice (4). In human patients with rheumatoid arthritis, etanercept in combination with methotrexate also ameliorates arthritis by reversing the Th1/Th2 ratio (5).

As a critical component of the immunologically mature T population, CD4^+^CD25^+^ regulatory T cells (Tregs) play crucial roles in maintaining self-immune tolerance and tuning down unwanted immune reactions, such as the inhibition of CD4^+^/CD8^+^ T cell activity and the mediation of the conversion of Th1 response into Th2 response (6,7). The pathogenic progression of arthritis is often associated with the dysfunction or the percentage change of peripheral Tregs (8). In acute gouty arthritis (AGA), the decrease of the Treg/Th17 ratio has been linked to the development of inflammation in joints (9). Tregs can also function to regulate the imbalanced Th1/Th2 responses under inflammatory conditions (10,11). Therefore, targeting Tregs has been proposed as an attractive strategy to curb the pathogenic progression of inflammatory diseases.

MicroRNAs (miRNAs) are short RNAs with 22-24 nucleotides in length that bind to the 3′ untranslated region (3′ UTR) of target mRNAs and induce mRNA degradation or arrest the translation (12,13). miRNAs regulate a myriad of biological processes, including cell proliferation, differentiation, secretion of inflammatory cytokines, tumorigenesis, hematopoiesis, and antiviral innate immune responses (14). Recently, miRNAs have been implicated in the regulation of Tregs immunity (15,16). For example, recent studies provide evidence that miR‐155 and miR‐181a are tightly related to Treg function and proliferation in allergic rhinitis (AR) (17). Nonetheless, it is unclear whether miR‐181a is involved in the regulation of Tregs in arthritis.

TGF-β1 is a key cytokine in the modulation of T cell differentiation and the balance of immune response. TGF-β1 binds to the type I receptors and also interacts with type II receptors to generate a heteromeric complex, which initiates signaling cascades to activate the Smad transcription factors (TFs) (18,19), and the nuclear translocation of Smad TFs triggers the expression of the target gene (20). However, the relationship between miR‐181a and CD4^+^CD25^+^ Treg activation in the pathogenesis of AGA and whether TGF-β1 signaling is involved in the above process is not clear.

In this work, a solution of monosodium urate (MSU) crystals was injected into the joint tissue of mice to induce AGA, and we examined the miR‐181a expression following the induction of AGA.

## Material and Methods

### Experimental animals

We obtained the 6- to 8-week-old C57BL/6 male mice from the Institute of Laboratory Animal Sciences, Chinese Academy of Medical Sciences (China). The mice were raised in individual cages in a pathogen-free animal house at controlled temperature of 22°C with a 12-h light/dark cycle. The mice were allowed to access drinking water and food freely prior to the experiment. The experimental procedures were conducted following the guideline for the Care and Use of Laboratory Animals released by the National Institutes of Health (USA). All the procedures were approved by the Experimental Animal Care and Ethics Committee of Tongji Hospital, Tongji Medical College, Huazhong University of Science and Technology.

### Acute gouty arthritis model

The AGA model was induced with MSU crystals (Sigma, USA) that were added to the injection solution with 10% Tween 80 dissolved in normal saline. The mice were anesthetized with 10% chloroform and administered 100 μg/mL MSU at the right ankle joint, using a 1-mL syringe that was inserted at an angle of 45° (17). An equivalent amount of MSU-free injection solution was applied in control mice at the identical site (n=10 mice/group).

### Isolation of CD4^+^ T cells and CD4^+^CD25^+^ Tregs from mouse spleen

Isolation of CD4^+^CD25^+^ Tregs were purified from the spleens of mice using CD4^+^CD25^+^ Regulatory T Cell Isolation Kit for mice (Miltenyi Biotec, USA). The spleen was smashed in sterile PBS and the mixture was filtered using 40-μm cell strainer. Cell suspensions were stained with biotin-conjugated depletion cocktail antibodies (anti-CD8, anti-CD19, anti-CD11b, anti-CD11c, anti-Ly6G, anti-TER119), and anti-biotin microbeads. Non-CD4^+^ cells were retained on the MS column of a MACS separator (Miltenyi Biotec) by negative selection. The eluted cells contained CD4^+^ cell population. To further purify CD4^+^CD25^+^ Tregs, the CD4^+^ cell population was further stained with PE-anti-CD25 antibody and anti-PE microbeads. The cells were then passed through the column on the MACS separator, and the retained cells were eluted as CD4^+^CD25^+^ Tregs. The remaining cells were used as primary CD4^+^CD25^-^ T cells. Isolated primary T cells were cultured in RPMI-1640 medium supplemented with 10% FBS, 50 μg/mL gentamicin, 50 μM 2-mercaptoethanol, and interleukin (IL)-2 (10 ng/mL).

### FOXP3 and CTLA-4 staining and analysis by flow cytometry

CD4^+^CD25^+^ Tregs isolated from the spleen of mice (10^5^ cells) were subjected to the staining solution with 2 μg/mL PE-conjugated anti-CTLA-4 Ab (BD Biosciences, USA) or anti-mouse IgG isotype control to characterize surface CTLA-4. Stained cells were washed twice with PBS and then analyzed by flow cytometry. Foxp3 expression was analyzed by intracellular staining. Tregs were fixed in 1 mL fresh fixation/permeabilization working solution (FoxP3 Staining Buffer Set, BD Biosciences). Fixed cells were washed with PBS and then re-suspended in the staining solution containing 5 μg/mL FITC-labeled anti-Foxp3 antibody or IgG isotype control (eBioscience, USA). The stained cells were washed twice with PBS and then analyzed using FACSCalibur cytometer and the CellQust software (BD Biosciences).

### Western blotting

Western blot was performed using isolated Tregs from mouse spleen. Total protein was extracted from Treg cells using RIPA lysis buffer containing protease inhibitor cocktail (Thermo Fisher Scientific, USA). Cells suspended in RIPA buffer were lysed on ice for 10 min, and the lysates were centrifuged at 13,200 *g* for 10 min at 4^o^C. The supernatant containing total protein lysate was quantified by a BCA Protein assay kit (Beyotime Biotechnology, China). The protein (10 µg) was used for SDS-PAGE electrophoresis and then transferred onto the PVDF membrane. After blocking with 5% non-fat milk, the membrane was incubated with primary antibodies anti-TGF-β1, Smad2/Smad2, and P-Smad2/Smad2 (1:1000, BD Biosciences). The membrane was washed 3 times with TBST and then incubated with HRP-linked secondary antibody (1:2500; Cell Signaling Technologies, USA) at room temperature for 1 h. The protein bands were visualized using a Pierce ECL Western blotting substrate (Thermo Fisher Scientific) and photographed on a gel imaging system (Bio-Rad, USA). The densitometry analysis was performed with ImageJ software (USA).

### Viral transduction and transfection

Genchem Co. (China) was responsible for preparing miR-181a mimic and the lentivirus. For increasing the TGF-β1 expression level, TGF-β1-RNA expressing recombinant lentivirus was transuded into Tregs in line with the specific protocols: primary Tregs were activated with plate-coated anti-CD3 (1 μg/mL) and anti-CD28 (1 μg/mL) overnight, and an equal volume of viral supernatant containing 8 μg/mL polybrene was added to the cell culture. After 12-h incubation, cells were harvested by centrifugation at 1000 *g* for 1.5 h at 32°C and transferred to fresh RPMI medium supplemented with IL-2. For transfection, 100 nm miR-181a mimic was transfected into Tregs using Lipofectamine 3000 (Thermo Fisher Scientific), according to the manufacturer's instructions.

### Cell proliferation assay

Primary cells (1×10^5^/well) were seeded in 96-well flat-bottomed plates and cultivated in RPMI-1640 medium supplemented with 10% FBS, 50 μg/mL gentamicin, 50 μM 2-mercaptoethanol, and IL-2 (10 ng/mL). Cells were treated with different experimental conditions and after 48 h, CCK-8 solution (10 μL, Dojindo Molecular Technologies, Japan) was added into each well for 3-h incubation. The light absorption value (OD value) under each condition was recorded at 450 nm on a Synergy H1 microplate reader (USA).

### Cytokine measurement by ELISA

To analyze cytokine production, 100 ng/mL PMA and 1 μg/mL ionomycin (BD Biosciences) were used to stimulate T lymphocytes for 24 h. The IL-2, IL-6 IL-10, and TGF-β in the cell culture supernatant were determined using commercial ELISA kits (R & D Systems, USA). In brief, 100 μL supernatant was added to the antibody-coated plate for 1 h incubation. After a washing step to remove unbound material, a biotin-labeled detection antibody was added for 1-h incubation, followed by the addition of streptavidin-HRP. After washing, 100 μL chemiluminescent substrate reagent was added for signal development and the absorbance of the testing samples, and standards was measured at 450 nm using a microplate reader. The concentration of each cytokine was measured based on the linear regression of the standards.

### RT-qPCR

Trizol reagent (Thermo Fisher Scientific) was used to extract RNA from tissues and cells according to the instructions. The extracted total RNA was dissolved in DEPC water, and 1 μg of total RNA was used for cDNA synthesis by a Mir-X miRNA first-strand synthesis kit (Takara, Japan) following the manufacturer's protocol. The resulted cDNA was analyzed in a 7500 Real Time PCR System (Applied Biosystems, USA) using SYBR¯ Green (Ampliqon, Denmark). Finally, the 2-ΔΔCt method was used to analyze the relative expression level, and the U6 gene was used as the internal reference gene. All primer sequences were synthesized by Shanghai Sangon Biotechnology Co., Ltd. (China): miR‐181a, F: 5′-GCTATCAGGTGTACTCAGATATG-3′; R: 5′-CTCAACGCACAGACGTGTC-3′; U6 gene primer: F: 5′-CACGAATTTGCGTGTCATCCTT-3′; R: 5′-GTGTAA CACGTCTATACGCCCA-3′.

### H&E staining

Hematoxylin and eosin (H&E) staining was performed using H&E Stain Kit (Abcam, UK). The deparaffinized/hydrated joint tissue section was incubated in hematoxylin (Mayer's solution) for 5 min. The section was rinsed twice with distilled water and then incubated with Bluing Reagent for 3 min. After washing with distilled water, the section was dehydrated in absolute alcohol and stained with eosin Y solution for 2 min. The section was rinsed using absolute ethanol and mounted on a slide for microscopic observation.

### Statistics

All data are reported as means±SD. All the experiments were repeated three times. Statistical differences between two groups were compared using unpaired Student's *t*-tests. Comparisons among multiple groups were analyzed using one-way analysis of variance (ANOVA). Comparisons of data at multiple time points were examined using two-way ANOVA. The SPSS 20.0 software (IBM, USA) was used. Differences with P<0.05 (two-sided) were considered to be statistically significant.

## Results

### Expression level of miR-181a was increased in Tregs isolated from AGA mice

**Figure 1:**
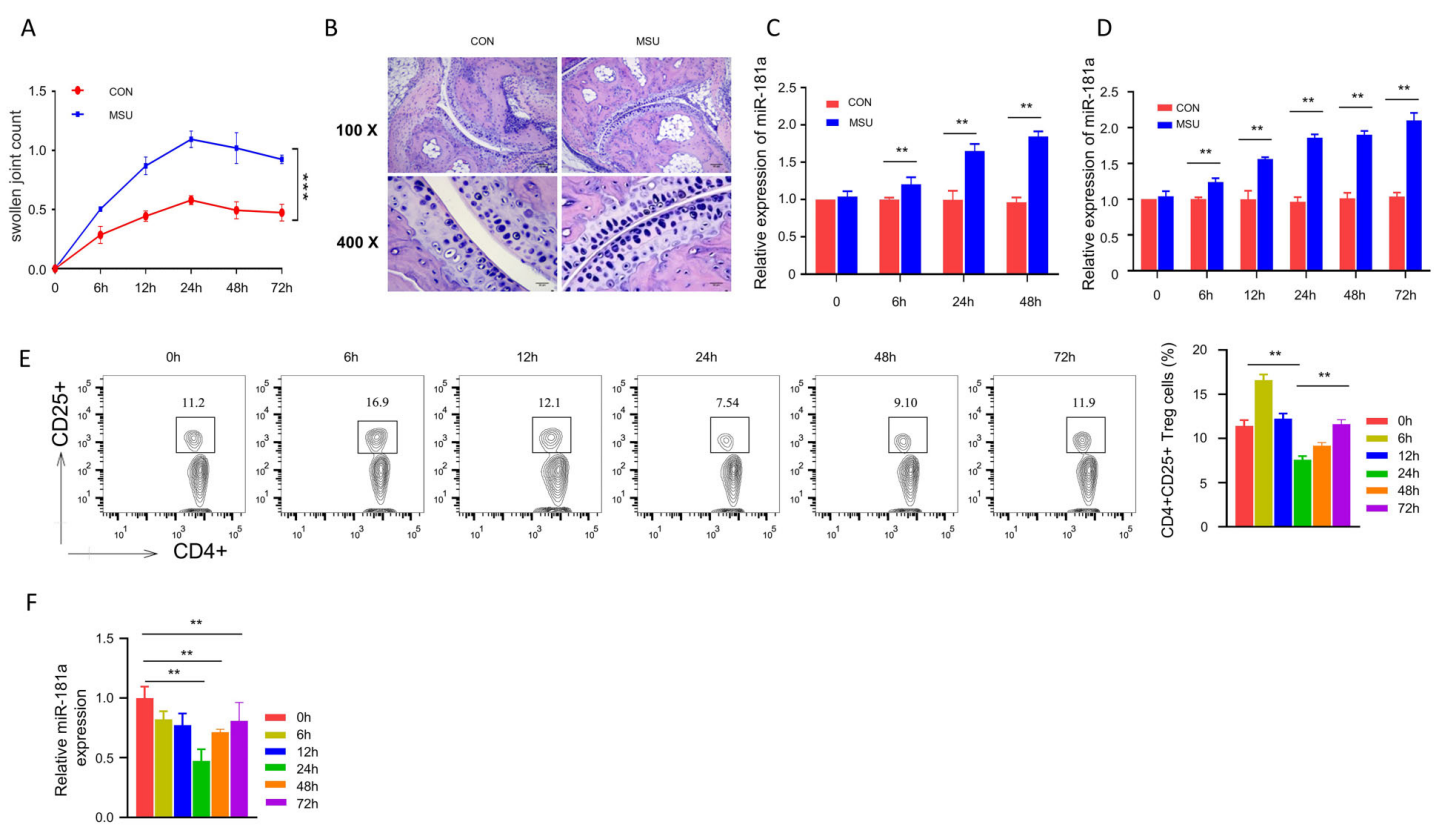
Expression levels of miR-181a in Tregs of acute gouty arthritis (AGA) mice (10 mice per group). **A**, The clinical scores of ankle joint in monosodium urate (MSU) crystal-induced mice and control (CON) mice were monitored for 72 h after induction. **B**, Massive inflammatory cell infiltration was detected within synovial tissues at 12 h by H&E staining (scale bars: 80 μm in upper panels and 20 μm in lower panels). **C**, Level of miR-181a was measured in joint tissues of MSU and CON mice. **D**, Expression of miR-181a was measured in Tregs isolated from both groups. **E**, Percentage of CD4^+^CD25^+^ Treg cells in total CD4^+^ T cells was measured by flow cytometry at different time points in MSU group. **F**, miR-181a expression level was quantified by qRT-PCR in Tregs at different time points after AGA induction. Data are reported as means±SD. **P<0.01; ***P<0.001 (ANOVA).

The ankles of mice in control and experimental groups were monitored for their clinical manifestations for 72 h after the injection. The clinical scores of ankle circumference were determined as the swelling index. The joint swelling in the MSU-injected experimental group increased significantly, which peaked at 24 h post-injection. There was mild swelling in the control group, and the joint swelling index was significantly higher in the MSU group (Figure 1A). At 12 h, H&E staining of the joint tissue showed that there was massive inflammatory cell infiltration within synovial tissues especially in the MSU group (Figure 1B). Together, these data indicated that MSU injection caused severe inflammation in the joint tissues.

The injection of MSU significantly upregulated miR-181a expression level at 24 and 48 h (Figure 1C). In addition, Tregs from AGA-induced mice showed a gradual increase in miR-181a expression level compared to the control group (Figure 1D). The percentage of Treg in CD4^+^ T cells also showed a sharp increase at 6 h, which gradually decreased at 24 and 48 h (Figure 1E). Additionally, miR-181a expression in Tregs showed a significant reduction at 24, 48, and 72 h after AGA induction (Figure 1F).

### miR-181a overexpression promoted TGF-**β**1/Smad signaling activation in Tregs

**Figure 2:**
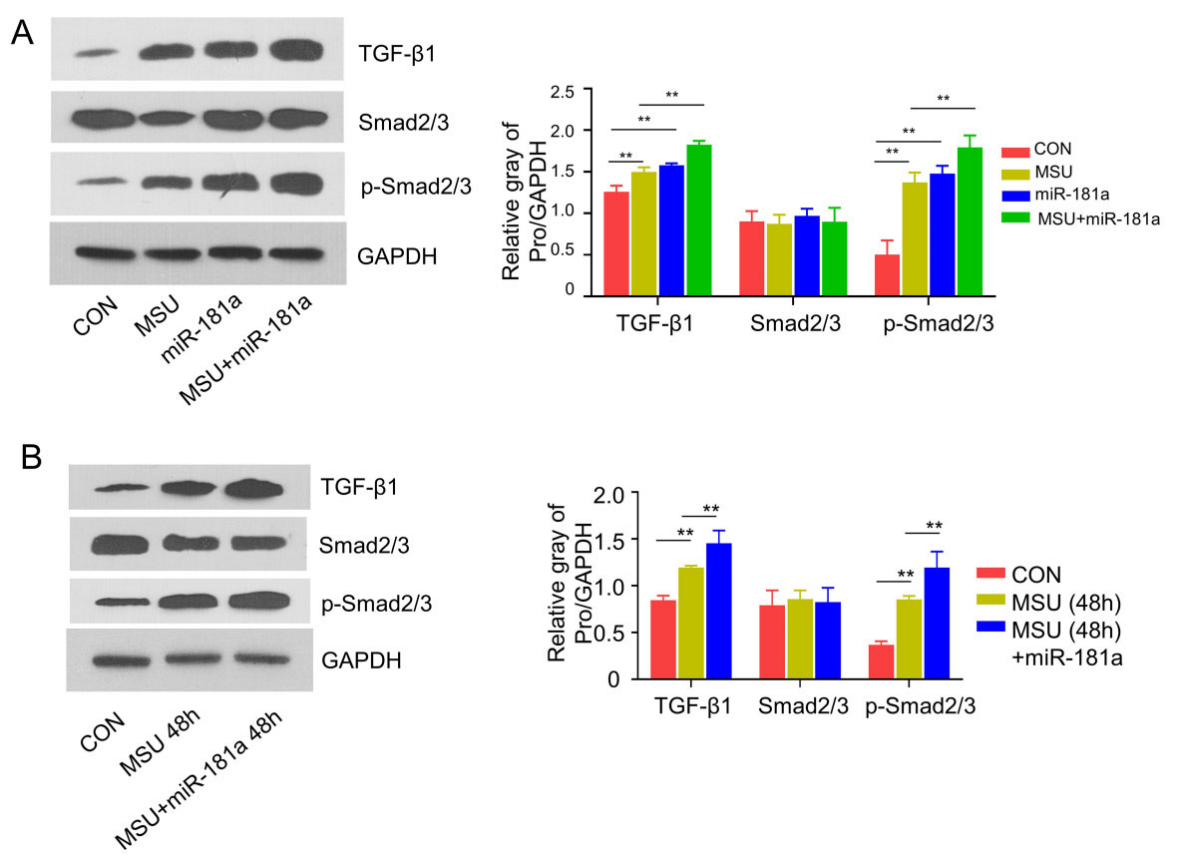
miR-181a overexpression promotes TGF-β1/Smad signaling activation. **A**, Protein levels of TGF-β1, Smad2/Smad3, and p-Smad2/Smad3 were measured in Tregs from control (CON) mice and monosodium urate (MSU)-induced mice at 24 h, in the presence or absence of miR-181a mimic. **B**, Protein levels of TGF-β1, Smad2/Smad3, and p-Smad2/Smad3 were measured in Tregs from both groups at 48 h, in the presence or absence of miR-181a mimic. Data are from three independent western blots. Data are reported as means±SD. **P<0.01 (ANOVA).

TGF-β1 signaling is a critical signaling pathway in Treg differentiation, and the activation of TGF-β1 signaling relies on the phosphorylation of transcription factors such as smad2/smad3. In total CD4^+^ T cells isolated from AGA mice (MSU group), the expression of TGF-β1 and the phosphorylation level of Smad2/Smad3 showed a significant increase compared to the Tregs from control mice. The transfection of miR-181a mimic significantly increased the activation level of TGF-β1 signaling in the presence or the absence of MSU crystals injection (Figure 2A). Similar results were observed in CD4^+^CD25^+^ Tregs, which showed that the transfection of miR-181a mimic synergized with MSU to promote TGF-β1 level and the phosphorylation of Smad2/Smad3 (Figure 2B). Together, these results suggested that miR-181a overexpression promoted TGF-β1/Smad signaling activation in Tregs.

### Overexpression of miR-181a promoted Treg responses by activating TGF-**β**1

**Figure 3:**
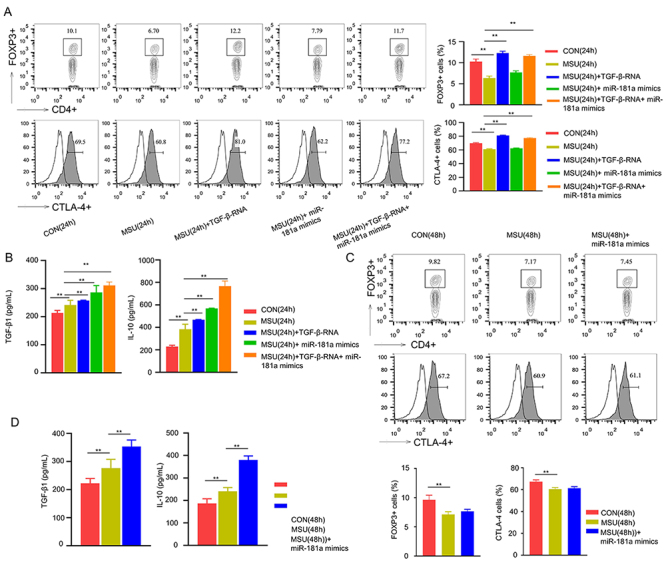
Overexpression of miR-181a enhances Treg responses via TGF-β1. **A**, CTLA-4 and Foxp3 expression levels were determined in cells from control (CON) mice and monosodium urate (MSU)-induced mice at 24 h, in the presence or absence of TGF-β and miR-181a overexpression. **B**, Upon MSU stimulation, increased IL-10 and TGF-β level was detected in Tregs isolated from MSU-induced mice at 24 h, and their expression level was further increased upon TGF-β and miR-181a overexpression. **C**, CTLA-4 and Foxp3 expression levels were determined in cells from both groups at 48 h, in the presence or absence of miR-181a overexpression. **D**, TGF-β and IL-10 were determined in Tregs isolated from both groups at 48 h, in the presence or absence of miR-181a overexpression. Data are from three independent experiments. Data are reported as means±SD. **P<0.01 (ANOVA).

To investigate the function of miR-181a in controlling the proportion of splenic Tregs, we analyzed the percentage of Tregs in total CD4^+^ cells isolated from control and MSU-injected mice at the early time point (24 h), in the presence of TGF-β-RNA overexpression (lentiviral transduction) or miR-181a overexpression (miR-181a mimic transfection). We performed intracellular Foxp3 staining and surface staining of CTLA-4A in different groups. As shown in Figure 3A, the percentage of Foxp3^+^ cells was decreased in the MSU group, which was rescued by the TGF-β overexpression. However, miR-181a mimic transfection did not increase the percentage of Foxp3^+^ Tregs (Figure 3A). Similarly, the percentage of CTLA-4^+^ cells in CD4^+^ population could also be increased by TGF-β-RNA overexpression, but not by miR-181a overexpression (Figure 3A). These data indicated that miR-181a did not affect Treg differentiation.

In Tregs isolated from MSU-injected mice, increased IL-10 and TGF-β expression was detected compared to the Tregs from control mice (Figure 3B). Interestingly, both TGF-β1 and miR-181a overexpression significantly promoted the level of IL-10 and TGF-β (Figure 3B). We further analyzed the percentage of Tregs in total CD4^+^ cells at the late time point (48 h). Similar to the results at 24 h, miR-181a overexpression did not affect Treg differentiation (Figure 3C). However, miR-181a overexpression significantly promoted the production of IL-10 and TGF-β in Tregs (Figure 3D). Together, these results suggested that miR-181a could regulate TGF-β and IL-10 production of splenic CD4^+^CD25^+^ Tregs via TGF-β1 activation.

### miR-181a overexpression enhanced the suppressive activity of Tregs

**Figure 4:**
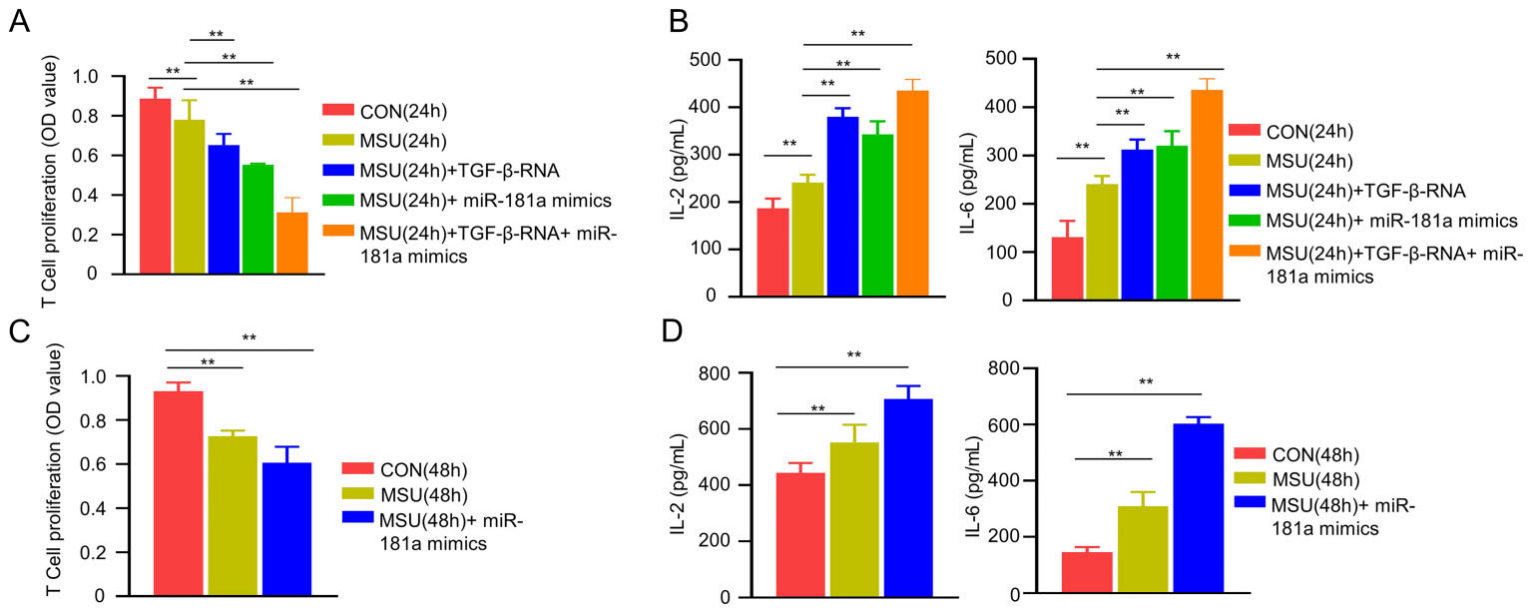
miR-181a regulates the suppressive activity of Tregs. **A**, CD4^+^CD25^+^ Tregs isolated from control (CON) or monosodium urate (MSU)-induced mice at 24 h were co-cultured with CD4^+^CD25^−^ T lymphocytes, in the presence or absence of TGF-β1 and miR-181a overexpression. The proliferation of CD4^+^CD25^−^ T lymphocytes was determined by CCK-8 assay. **B**, Interleukin (IL)-2 and IL-6 cytokine levels were measured in Tregs under the conditions described in (**A**). **C**, CD4^+^CD25^+^ Tregs isolated from both groups at 48 h were co-cultured with CD4^+^CD25^−^ T lymphocytes, in the presence or absence of miR-181a overexpression. The proliferation of CD4^+^CD25^−^ T lymphocytes was determined by CCK-8 assay. **D**, IL-2 and IL-6 cytokine levels were measured in Tregs under the conditions described in (**C**). Data are from three independent experiments. Data are reported as means±SD. **P<0.01 (ANOVA).

One of the major functions of Tregs is to suppress the proliferation of CD4^+^ effector T cells. We therefore examined whether miR-181a modulates the suppressive activity of Tregs by co-culturing CD4^+^CD25^+^ Tregs with CD4^+^CD25^−^ T lymphocytes at a 1:1 ratio. Tregs isolated from MSU-treated mice at 24 h showed stronger suppressive activity on D4^+^CD25^−^ T lymphocytes, and TGF-β1 and miR-181a overexpression significantly augmented the inhibitory effect of Tregs (Figure 4A). Further, Tregs isolated from MSU-treated mice at 24 h produced more IL-2 and IL-6 than the Tregs isolated from control mice, and both TGF-β1 and miR-181a overexpression further promoted the production of IL-2 and IL-6 (Figure 4B). Similar results were observed from Tregs isolated from control and MSU-treated mice at 48 h (Figure 4C and D). Together, these results suggested that miR-181a enhanced the suppressive activity of Tregs by modulating cytokine production such as IL-2.

## Discussion

The changes of the percentage of Tregs in CD4+ T lymphocytes and their suppressive activity can influence the progression of inflammation and autoimmunity. Understanding the mechanisms underlying Treg differentiation and activity regulation could provide novel insight into the modulation of inflammatory diseases (21). It is well-known that miR‐181a is implicated in the proliferation and progression of various cancers (22-25). Previous studies showed that Treg population could be reduced under certain pathogenic conditions such as systemic lupus erythematosus (26) and rheumatoid arthritis (27), but whether microRNA is implicated in the regulation of Treg population and activity under AGA pathogenic condition is unknown. In addition, it remains to be determined whether TGF-β1 signaling participates in the AGA pathogenesis.

In this study, we established an AGA mouse model by injecting MSU crystals into the ankle joint of mice. Our data showed that MSU injection caused massive inflammatory cell infiltration (monocytes and neutrophils) in the swollen joint synovium, which is the cause of the pain and swollen joints in the patients of AGA (28). Our data and another study suggest that AGA animal models could shed light on the mechanisms underlying the inflammation and pain in the joint tissues of AGA patients (29). Interestingly, our data revealed that miR-181a expression was increased in the CD4^+^CD25^+^ Tregs isolated from MSU crystals-induced AGA mice. In addition, miR-181a expression was correlated with the changes of the percentage of splenic CD4^+^CD25^+^ Tregs *in vivo* at 24 and 48 h after MSU crystal injection. Together, our data suggested that miR-181a upregulation may affect Treg differentiation or Treg activity in AGA. This result seems to be consistent with a previous report that miR-181a is implicated in the regulation of the differentiation and function of regulatory T cells in allergic rhinitis (17). However, further studies are required to delineate the mechanism underlying the upregulation of miR-181a in Tregs of AGA model.

Our results further demonstrated that miR-181a may regulate Tregs by modulating TGF-β1/Smad signaling activation. TGF-β1 signaling pathway is critical in the regulation of Treg differentiation and activity in peripheral organs (30,31). Interestingly, previous studies have widely reported that miR-181a could regulate TGF-β expression or activity in different cell models, such as cancer cell (32), osteoblast (33), and fibroblast (34). Although their regulatory mechanisms may be different, these data suggest that miR-181a is connected to the TGF-β pathway. How miR-181a regulates TGF-β expression in Tregs remains to be further investigated.

Under inflammatory conditions such as arthritis, the CD4^+^CD25^+^ Treg population is increased (35,36). In our model of AGA, we found a sharp increase of Treg proportion at an early time point (6 h), but a reduction at later time points (24 and 48 h) after AGA induction. These data suggest that acute induction of AGA may first induce expansion of the Treg population followed by an exhaustion of Tregs. Previous studies also indicate that microRNA-mediated feedback and feed forward loops could regulate the immune system (37). Our data showed that, unlike TGF-β signaling, miR-181a overexpression did not affect the expression of Foxp3 and CTLA4 levels in Tregs. Instead, miR-181a overexpression promoted the suppressive activity of Tregs, which was accompanied by the elevated IL-2 production. It is well-known that Tregs have a high affinity for IL-2, and IL-2 is required for the optimal activity of Treg function (38,39). Therefore, our data suggested that miR-181a may regulate Treg activity by modulating IL-2 expression. Further efforts are required to unveil how miR-181a controls IL-2 expression in Tregs.

In summary, this study suggested that miR-181a expression was tightly related to the activity of TGF-β1 in Tregs of an AGA model, and the upregulation of miR-181a could affect the immune functions of Tregs. Our study further indicated that miR-181a regulated the activity of Tregs cell via targeting the TGF-β1/Smad pathway and IL-2 synthesis. The above results uncovered a novel role of miR-181a in Treg immunity of the AGA model.
